# Editorial: Deciphering signaling pathway interactions in tissue homeostasis

**DOI:** 10.3389/fcell.2025.1677790

**Published:** 2025-08-12

**Authors:** John T. Hancock

**Affiliations:** School of Applied Sciences, University of the West of England, Bristol, United Kingdom

**Keywords:** cancer, cell signaling, esophageal epithelium, inflammation, immune

Cells, tissues and organisms need to maintain levels and balances of metabolites and functional systems whilst constantly being under the influence of external pressures, a process known as homeostasis. Cells need to achieve this to survive and prosper, and such control is determined by the cell signaling pathways involved. There have been many reviews on the topic, for example, showing the range of cell types and tissues that have been studied, such as B cells (Woodland et al., 2006), T cells (Sprent et al., 2008), and liver cells (Stanger, 2015). To maintain cellular homeostasis, cells need to perceive a range of extracellular signals, and coordinate these into an appropriate response. This involves extracellular signals such as cytokines, oxygen levels and ATP, as well as cell surface receptors, intracellular signaling pathways and intracellular organelles, in a process which has been dubbed the homeostatic circuit (Meizlish et al., 2021). This is an extremely complex, but instrumental system, to ensure cellular, and organismal, longevity.

Many systems need to be controlled in a balanced manner. A good example is the production and removal of redox molecules, such as reactive oxygen species and nitric oxide, and these are interconnected in more overarching signaling networks which controls homeostasis (Zhang et al., 2022). The immune system too needs to be controlled in a careful manner, and if this goes awry it can lead to disease states. For example, see reviews by Swirski and Nahrendorf (2018), Antonangeli et al. (2021), and Slominski et al. (2025).

It was therefore timely that a Research Topic attempted to bring together work that was focused on the understanding of cellular and tissue homeostasis.

Four papers were published in the Research Topic. Grommisch et al. gave a mini review of the state of play with the esophageal epithelium, an important tissue which allows food transport but also gives protection from erroneous ingress. They point out that the work on mice does not give a full understanding of the situation in human tissues, and therefore compared the two. They go on to discuss the model systems which are currently available to further human studies, information which would be useful for anyone taking this topic forward. Lastly, Grommisch et al. look at the signaling networks involved in these cells, with a view to getting a better understanding of disease states and treatments. Pathways discussed include sonic hedgehog, retinoic acid signaling, and the BMP and WNT pathways. In their outlook section, they highlight the use of hPSC-derived esophageal mesenchymal cells as a major step forward to understand the biology of the esophageal epithelium, including its development and disease states.

Park et al. reported on signaling pathways in brown adipocytes. Their focus was on malate-aspartate shuttle (MAS), and in particular the enzymes glutamic-oxaloacetic transaminases (cGOT1, mGOT2) and malate dehydrogenases (cMDH1, mMDH2). This article followed on from a previous report by the same group, where GOT1 levels were increased in brown adipocytes during cold exposure (Park et al., 2024). This involved the signaling pathway from the β-adrenergic receptor (βAR), which controlled transcription mediated by cAMP and protein kinase A (PKA). In the present paper, they go on to look at the stability of GOT1, which they say is mediated by pathways involving βAR and serum- and glucocorticoid-inducible Kinase 1 (SGK1). This prevents ubiquitination of GOT1 by E3 ubiquitin ligase RNF34, and hence GOT1 is not destroyed by the proteasome. They could show this by using genetically modified forms of SGK1, as well as the use of pharmacological treatments. Such work shows how these pathways can lead to metabolic adaptation during an externally applied environmental pressure on these cells.

The signaling pathways involved in systemic disorders were the focus of a review by Yang et al. They point out that stressed or damaged cells can trigger the immune response and chronic inflammation, which is mediated by the release of High mobility group box-1 (HMGB1). NOD-like receptor (NLR) family pyrin domain-containing 3 (NLRP3) is involved in caspase-1 activation, with the downstream release of pro-inflammatory cytokines, including IL-1β and IL-18. Therefore, understanding these pathways, and targeting their action, may be fruitful for the control of long-term and systemic inflammatory conditions. This review covers the structures and the functions of HMGB1 and NLRP3, and then goes on to discuss their actions and influence in inflammatory disease, including that which involves the neuronal, respiratory, digestive, urinal, genital, motor, immune, and circulatory systems. They then discuss therapeutic options which can be considered to treat long-term immune-based conditions.

Lastly in this Research Topic, Brutscher and Basler discuss in their mini review Toll/NF-κB signaling in tissue homeostasis and cancer, with a focus on imaginal tissue of *Drosophila*. They give an overview of the Toll/NF-κB pathway, and then go on to discuss how this functions in the homeostasis of imaginal tissues. Here, the discussion covers the regulation of cell competition, the role of cell death in development, and the control of tumorous growth. This leads nicely to the discussion of the misuse of Toll/NF-κB signaling pathways in tumor development and progression.

Although this is a small Research Topic of articles, they do bring together a range of tissues and cells, and signaling systems. This further highlights the need for an integrated approach to our understanding of a range of signaling pathways and how they come together to control homeostasis in cells, tissues and in a systemic manner in organisms, as well as how such pathways may be targeted for future disease treatments.
